# The impact of informal and formal care disruption on older adults’ psychological distress during the COVID-19 pandemic in UK

**DOI:** 10.1016/j.ehb.2023.101242

**Published:** 2023-04

**Authors:** Cinzia Di Novi, Gianmaria Martini, Caterina Sturaro

**Affiliations:** a European Commission, Joint Research Centre (JRC), Ispra, Italy; b Department of Economics, University of Bergamo, Italy

**Keywords:** Informal care, Formal care, Mental health, Elderly, Disruption, COVID-19

## Abstract

This paper investigates how formal and informal caregiving disruptions-due to the U.K. government’s non-pharmaceutical interventions (NPIs) aimed at reducing transmission of the SARS-CoV-2 virus-may have affected the likelihood of psychological distress among older individuals. We model the association between disruption of formal and informal care and mental health of the elderly during the first wave of the COVID-19 pandemic using a recursive simultaneous - equation model for binary variables. Our findings reveal that public interventions, which are most essential for reducing the pandemic spread, influenced the provision of formal and informal care. The lack of adequate long-term care following the COVID-19 outbreak has also had negative repercussions on the psychological well-being of these adults.

## Highlights

• We investigated how formal and informal caregiving disruptions due to COVID-19 pandemic affected elderly's psychological distress in U.K.
• We used a recursive simultaneous equation model for binary variables.
• The disruption of long-term care following the COVID-19 outbreak had negative repercussions on the psychological well-being of the elderly.

## 1 Introduction

The first national lockdown to mitigate the transmission of COVID-19 in the U.K. was introduced on March 23, 2020 and remained in place until July 4, 2020. During the lockdown the government imposed national restrictions and required all those who could to work from home, closed all but essential shops, and advised the population to stay at home and limit contact with other people outside of their households. Moreover, the U.K.’s National Health Service (NHS) identified specific “clinically vulnerable” individuals thought to be at higher risk of severe COVID-19 complications and related deaths, and strongly advised them to stay home and avoid all face-to-face contact. The entire elderly population, regardless of individual medical conditions, was also considered clinically vulnerable and advised to stay home as much as possible (Public Health England, 2020, Cabinet Office, 2020).

Although effective in preventing a further dissemination of COVID-19, these interventions were immensely disruptive to people’s social connections and had potential repercussions on sectors with high direct face-to-face contacst-e.g., the healthcare industry and social services (Bu et al., 2020). Vulnerable groups such as older people encountered unique and remarkable challenges in coping with their care needs without leaving their homes (Age U.K, 2020).

In the U.K., elderly support is dependent upon a combination of informal and formal care: statutory-source community care and social services, privately paid care workers, neighbors, friends, and family members; Vlachantoni et al. (2015); Maplethorpe et al. (2015)). The strict restrictions introduced by the U.K. government, together with the reorganization of the healthcare system at all levels, produced a disruption in both types of caregiver activities (Topriceanu et al., 2021).

Previous literature on the effects of the COVID-19 pandemic on long-term care (LTC) has paid significant attention on the limited availability of formal care services during the pandemic that have placed additional burdens on family caregivers in terms of objective (i.e., hours spent on caring) and subjective burdens (i.e., mental health and quality of life) (see, for instance, Maccora et al., 2020; Rodrigues et al., 2021; Tsapanou et al., 2021; Leggett et al., 2021; Monteiro et al., 2022; McGarrigle et al., 2022; Costi et al., 2023).^2^ However, an investigation into the effects of COVID-19 and its accompanying control measures on formal and informal care disruptions, on elderly unmet care needs, and health-related outcomes (i.e., physical, and mental health) has remained relatively scant.

Relying on data from the Understanding Society COVID-19 Survey (April 2020) during first the COVID-19 wave across the U.K, Evandrou et al. (2020) provided the first descriptive evidence on informal care disruptions affecting the elderly during this time. The authors investigated the extent of support received by older people from family, friends, and neighbors in the first period of the lockdown. According to their findings, a significant proportion of older people received an increased level of help (ranging from shopping, dressing, meal preparation, assisting with online or internet access, gardening, or house repairs) from those who had provided care to them before the outbreak or from new caregivers. This was especially the case among those living alone or with a partner aged 70 and over. However, Evandrou et al. (2020) also showed that a smaller group of frail elderly people with difficulties in performing key activities of daily living suffered from an informal care disruption and received less care and support during the lockdown compared to the pre-COVID-19 outbreak period. This evidence raised the specter that a group of older vulnerable individuals might not have received an adequate level of social care during the lockdown.

Tur-Sinai et al. (2021) investigated how the initial outbreak influenced the supply of formal and informal care among the elderly in need in 23 European countries and Israel by using data from the Survey of Health, Ageing and Retirement in Europe (SHARE Corona Survey), again adopting a descriptive approach. According to their findings, in the first months of the outbreak, informal care appeared to be more resilient than formal care services; indeed, a significant proportion of older adults in European countries continued to receive informal help, enjoying an increase in the amount of care from children, neighbors, friends, and colleagues, while informal help from other relatives decreased. Alternatively, older adults encountered great difficulty in obtaining formal help from professional caregivers.

Brugiavini et al. (2022) investigated whether the disruption of elderly parent–adult child contacts due to social distancing restrictions, which characterized European countries during the first wave of the pandemic, increased symptoms of depression in the elderly, using the eighth wave of the SHARE and the SHARE Corona Survey. They adopted a joint model of parent-child contact disruption and mental health issues, estimated by using a recursive bivariate probit model. Their findings showed that interventions deemed essential to reduce the spread of the pandemic, including physical distancing and other epidemiological control measures (e.g., stay-at-home orders, travel restrictions, and so forth), disrupted some personal parent–child contacts, with negative consequences on the elderly parents’ mental health.

To the best of our knowledge, no studies have been conducted on the connection between disruption of formal care and its potential impact on the elderly population’s mental health, nor on the inter-relationship between formal and informal care disruptions due to lockdown restrictions and older adults’ mental-health deterioration in the U.K context. This paper aims to fill this gap by providing additional insights regarding the short-term consequences of mental health care disruptions to the elderly during the COVID-19 outbreak on the elderly. The empirical evidence provided by this paper may shed light on the importance of designing public policies to contain pandemic crises with the realization that some population groups are more affected than others. Hence, these groups need different social restrictions from those imposed on the general population since they may suffer more from the consequences of isolation and reduction in social contacts (Gulland, 2020, Carers UK, 2020).

For the purposes of our study, we used data from the U.K. Household Longitudinal Study (U.K.HLS) Understanding Society (waves #9 and #10), and the COVID-19 Survey (wave #1, April 2020). Following Brugiavini et al. (2022), we attempt to study the complex relationship between informal and formal care disruption and elderly psychological well-being. As such, we used a simultaneous equation model for binary variables. Specifically, we constructed a joint model of informal care and formal care disruption and mental health conditions that considers an individual’s unobserved heterogeneity that may characterize this relationship.

Our findings show that the disruption of informal and formal support represents a significant risk factor for psychological well-being in older adults and increases their risk of depression.

## 2 Data

This study uses individual-level data from the U.K. Household Longitudinal Study (U.K. HLS), Understanding Society, a nationally representative panel study of the British population. For the HLS, sample members living in the U.K. were interviewed annually since 2009 with the aim of recruiting over 100,000 individuals in 40,000 households. The first wave of the study and data collection period spanned two years and thus wave #1 ran from 2009 to 2011, wave #2 from 2010 to 2012, and so on. Since April 2020, a subsample of participants from the U.K. HLS survey have been interviewed each month, and they completed short web surveys that focused on the impact of the COVID-19 pandemic. The short web surveys covered the changing impact of the pandemic on the welfare of individuals and households. Each month, participants completed one survey that included core content designed to track changes alongside variable updated content as the coronavirus situation developed. Core modules included detailed information on household composition, coronavirus illness, long-term health conditions management, mental health measures, loneliness, and employment. Individuals were identified by a personal unique identifier that remained for all waves and could be used to link respondents’ information across different waves (Institute for Social and Economic Research, 2020).

The integrated data set used for this analysis is the result of matching wave #9 (2017–2019) and #10 (2018–2020) of the main survey and the first month of the COVID-19 wave (April 2020). This data set provided us the opportunity of gathering information related to the COVID-19 outbreak and the years before it.

After correcting for missing values, the sample included 3721 individuals. In this paper, we focused specifically on individuals aged 65 and over and found that the COVID-19 pandemic took a heavy toll on their physical as well as mental health. The measures adopted by the U.K. government regarding social distancing and isolation to protect the elderly from risk of infection often resulted in social isolation and loneliness (to which older adults are more vulnerable because of their functional dependency) that in turn might have increased their likelihood of depression (Banerjee, 2020).

## 3 Empirical strategy

### 3.1 Dependent variables

As previously discussed, the main aim of this study was to investigate the potential effects of informal and formal care disruptions on the mental health deterioration of older people in the U.K during lockdown restrictions intended to curb the COVID-19 spread.

The first step toward a full understanding of this effect required a complex model that considered the simultaneous relationships between informal and formal care disruption and older individuals’ psychological well-being. Following Brugiavini et al. (2022), we employed a simultaneous equation model for binary variables. We constructed a joint model of informal and formal care disruption and mental health outcomes that we estimated through a recursive multivariate probit model that considers individuals’ unobserved heterogeneity that may characterize these relationships (see Subsection 3.2).^3^ Thus, we identified two classes of dependent variables: informal and formal care reception and mental health outcomes—i.e., older individuals’ psychological distress. To measure individuals’ psychological distress, we used the 12-item Generalised Health Questionnaire (GHQ-12), which is one of the most widely used screening tools for psychological distress that has been validated for epidemiological studies (Goldberg et al., 1997). The GHQ-12 was collected in all waves of the U.K. HLS Understanding Society to date and included in the Understanding Society COVID-19 Survey. Each one of its 12 items regarding symptoms, feelings, or behaviors is answered on a four-category Likert scale ranging from “*not at all*” to “*much more than usual*”: categories 1 and 2 (“*not at all*,” “*no more than usual*”) were scored as 0, and categories 3 and 4 (“*rather more than usual*,” and “*much more than usual*”) were scored as 1.^4^ Finally, the scores from the 12 items were added to obtain an overall score. The measure attained in this way is called GHQ-12 Caseness and respondents scoring 3 or more (out of a possible total of 12) are likely to be experiencing anxiety and/or depression (Cox et al., 1987). In line with the literature, GHQ‐12 Caseness > =3 is used as the threshold to define our dichotomous outcome variable (Lindkvist and Feldman, 2016, Aalto et al., 2012, Holi et al., 2003).^5^

To generate a variable that accurately measures the disruption of informal care, we considered the following questions included in the first wave of the Understanding Society COVID-19 Survey: “*Thinking about the last 4 weeks, did you receive support from family, neighbors or friends who do not currently live in the same house/flat as you*?” (with “yes” or “no” answer options), and “*Thinking back to earlier this year, before the outbreak of the coronavirus pandemic. How has the help and support you receive from family, friends or neighbors who do not live in the same house/flat as you changed*?” (Response options included: “1. There has been no change; 2. I receive more help from some people who previously helped me; 3. I receive less help from some people who previously helped me; 4. I currently receive help from family, friends or neighbors who did not previously help me”). To capture a potential disruption in informal care, we constructed a binary variable that takes the value of 1 if respondents reported they had not received informal care in the last 4 weeks before the interview (from non-cohabiting family members, neighbors, or friends), but they had received help before the outbreak, or if they had received less help from certain people who previously helped them, and 0 otherwise (if they had received support in the last 4 weeks before the interview, or if they had not received support in the last 4 weeks before the interview, but there has been no change with respect to the pre-outbreak period).

In reference to formal care (i.e., community health and social care services), the Understanding Society COVID-19 Survey asked respondents “*in need*” of formal care to report whether they had received help with personal care/medications/shopping/cooking/cleaning/wound dressing/injections from someone visiting them at home regularly before the pandemic restrictions.^6^ The answers ranged from 1 to 4, specifically: “1. Yes, as before; 2. Yes, but with reduced support; 3. Yes, with increased support; 4. No.” We constructed a binary indicator that takes a value 1 if respondents, who needed formal care, reported they had experienced a reduction in community health and social care services in 2020, or they did not receive any services compared to the pre-pandemic period, and 0 otherwise.

According to Evandrou et al. (2020) a relatively low proportion of the elderly reported a disruption in informal care and formal care received during the first COVID-19 wave. Indeed, about 4% of the elderly in our sample experienced a disruption in informal care received, while about 3% reported a disruption in formal care.

### 3.2 Estimation method

Identifying an association between formal and informal care disruption and the mental health of the elderly may be complicated by the presence of endogeneity. Older individuals’ isolation, resulting from the U.K. government restrictions to contain the virus, might have increased the risk of depression while simultaneously influencing access to formal and informal support (Cacioppo et al., 2006, Holt-Lunstad et al., 2010). In this application, the situation is further complicated because both formal and informal home care may be simultaneously determined (van Houtven & Norton, 2004). Indeed, receiving informal care may be correlated to unobserved health characteristics or to unobserved preferences for care that are likely to influence the demand for formal care (Charles and Sevak, 2005, Bonsang, 2009). Moreover, the probability of accessing formal care and informal care may have been influenced by the pandemic. As such, we estimated the model using a recursive multivariate probit design. The recursive structure of the multivariate probit model builds on a structural-form equation that determines the probability of the onset mental health conditions and two reduced-form equations: one for the potentially endogenous dummy variable measuring the disruption of informal care received; and the other for the potentially endogenous dummy variable measuring the disruption of formal care.

Hence, we identified two classes of dependent variables: care disruption—namely, formal, and informal care—and health outcome (i.e., the dummy indicator for individuals’ mental health as measured by the GHQ-12 Caseness score). In the structural equation for mental health, formal and informal care disruption are included as regressors.

We constructed and estimated a system of three equations with two reduced-form equations and one structural equation represented by the mental health equation. Thus:

$$y_{3i}^{*}=\beta_{3}^{\prime }x_{3i}+\varepsilon_{3i}=\delta_{1}y_{2i}+\delta_{2}y_{3i}+\alpha_{3}^{\prime }z_{3i}+\varepsilon_{3i}$$

(1) $$y_{2i}^{*}=\beta_{2}^{\prime }x_{2i}+\varepsilon_{2i}$$

$$y_{1i}^{*}=\beta_{1}^{\prime }x_{1i}+\varepsilon_{1i},$$

where ***x***_*li*_ (with l = 1, 2) and ***z***_*3i*_ are vectors of exogenous variables, $\beta_{1}^{\prime },\;\beta_{2}^{\prime }$ and $\alpha_{3}^{\prime }$ are parameter vectors, and $\delta_{o}$ (with o = 1, 2) are scalar parameters. The error terms distributed as multivariate normal are $\varepsilon_{\mathrm{hi}}$(with h = 1, 2, 3), each with a mean zero, and variance covariance matrix Σ. Σ has values of 1 on the leading diagonal and correlations $\rho_{\mathrm{jk}}=\rho_{\mathrm{kji}}$ on the off-diagonal elements (where $\rho_{\mathrm{jk}}$ is the covariance between the error terms of equation *j* and *k*).

In the abovementioned setting, the exogeneity condition is stated in terms of the correlation coefficients, which can be interpreted as the correlation between the unobservable explanatory variables of the different equations. All equations in system (1) can be estimated separately as single probit models only in the case of independent error terms (i.e., the coefficient $\rho_{\mathrm{jk}}$is not significantly different from zero).

Conventionally, the identification of a recursive multivariate probit model has been based on exclusion restrictions to obtain a more robust identification of the parameters. Maddala (1983) proposed that at least one of exogenous variables (i.e., in the vectors ***x***_***1i***_ and ***x***_***2i***_) of the reduced-form equations is not included in the structural equation as an explanatory variable. However, more recent work by Wilde (2000) shows that identification is achieved even if the same regressors appear in all equations providing there is sufficient variation in the data (i.e., providing each equation contains at least one varying exogenous regressor). Nevertheless, this result is valid in the context of multivariate normal distribution, and, in the absence of additional instruments, identification strongly relies on functional form—i.e., normality of the stochastic disturbances, commonly referred to as identification by functional form (Li et al., 2019a, Li et al., 2019b). It is therefore common practice to impose exclusion restrictions to improve identification of the causal parameters$\delta_{1}$and$\delta_{2}$. These exclusion restrictions (instruments) should be causally linked to informal and formal care disruption and should affect individuals’ mental health only through their effects on informal and formal care disruptions. The instruments are discussed in detail in Subsection 3.3.

### 3.3 Exclusion restrictions

This subsection describes the exclusion restrictions that we adopted for both reduced-form equations.

#### 3.3.1 Disruption of informal care equation

The emergence of COVID-19 and the measures implemented by the U.K. government to curb its spread forced frail older people indoors and reduced opportunities to remain socially connected. In March 2020, a stay-at-home order was issued that banned all non-essential movements and contact with other people outside the household. This restriction had important repercussions on the continuity of the informal care provision mainly because (non-cohabiting) caregivers faced difficulties traveling to the homes of recipients. In a period characterized by stringent mobility restrictions, traveling a small geographical distance to provide help might have represented an important barrier to caregiving. Wave #9 of the Understanding Society Survey includes a question regarding which non-coresident relatives’ respondents are “*alive at the moment*.” Respondents with children living outside the household were then asked how long it takes them—door to door—to travel to their sons’ or daughters’ residences (aged 16 or over). If respondents reported they have more than one non-coresident child aged 16 or over, they were asked to think about the child with whom they have the most contact. Thus, we create a binary variable that takes the value of 1 if respondents lived more than 30 min travel time from their children (time taken by usual mode of transport) and 0 otherwise (the cut-off was chosen following Li et al., 2019a, Li et al., 2019b; Thomas and Dommermuth, 2020; Artamonova and Syse, 2021).^7^

We also include in the reduced-form equation for informal care disruption a binary variable that takes the value of 1 if none of the respondent’s friends live in his or her local area.

We gathered this information from wave #9 in the “Family Networks” and “Social Network” modules, respectively (that were not included in the most recent waves #10 and the COVID-19 Survey), by assuming that non-proximity with children and friends remained broadly constant over time.

#### 3.3.2 Disruption of formal care equation

While the U.K.’s NHS provides universal healthcare, the provision of publicly funded formal long-term care (LTC) services is based on a needs assessment (i.e., whether the potential care recipient can eat, wash, or dress without help) and means assessment (i.e., income that includes pensions, benefits, and assets), and it is a statutory responsibility of local authorities. In cases where care needs do not meet the criteria or financial means are above the threshold, formal care services should be privately purchased: individuals being cared for (or their family) pay all or most of the costs for their care.

In the last decade, the means test has become meaner, and the usage rate of social services has declined. Among those who must pay for themselves, cost was often cited as a reason for not seeking help (AgeUK, 2022). The pandemic further exacerbated this affordability challenge for many older households, and thereby increased their risk of care disruption (Phillipson et al., 2021).

The Social Care Module of the wave #9 of the Understanding Society Survey includes information about who usually manages payment for the care provider. We created a binary variable that takes the value of 1 if the respondents themselves paid for all formal pre-pandemic care services without any support from family, friends, or local authorities. We expect that those who did not receive any support in paying for the costs of services might have significantly suffered from worse care access and a higher probability of formal care disruption.

### 3.4 Other independent variables

Table 1 shows the other independent variables in the three equations model of (1), grouped into listed categories.

**Table 1:** Variables Name and Definition.

*Variables name*	*Definition*	*COVID-19 Survey wave/ U.K. H–S - Understanding Society wave*
***Dependent variables***		
Mental Health Conditions/ Psychological Distress 2020 (GHQ>=3)	1 if GHQ-12 Caseness items score is greater or equal than 3 reflecting deteriorations in mental health, 0 otherwise.	COVID-19 Survey wave #1
Formal Care Disruption	1 if respondent did not receive formal care or received reduced formal care with respect to period before COVID-19 outbreak, 0 otherwise.	COVID-19 Survey wave #1
Informal Care Disruption	1 if respondent experienced a decrease in the provision of care in the four weeks before the interview, with respect to the period before the outbreak of COVID-19, 0 otherwise.	COVID-19 Survey wave #1
***Independent variables***		
Age	continuous variable	COVID-19 Survey wave #1
Male	1 if male, 0 female	COVID-19 Survey wave #1
Rural	1 if lives in a rural area, 0 urban area	U.K. HLS - Understanding Society wave #10
England	1 if lives in England, 0 otherwise	U.K. HLS - Understanding Society wave #10
Wales	1 if lives Wales, 0 otherwise	U.K. HLS - Understanding Society wave #10
Scotland	1 if lives in Scotland, 0 otherwise	U.K. HLS - Understanding Society wave #10
Northern Ireland	1 if lives in Northern Ireland, 0 otherwise	U.K. HLS - Understanding Society wave #10
Living with partner	1 if lives with partner, 0 if alone	COVID-19 Survey wave #1
Lower education	1 if completed level of education is null or 1–2 of U.K. education system, 0 otherwise	U.K. HLS - Understanding Society wave #10
Medium education and other qualification	1 if completed level 3 of U.K. education system or other qualification, 0 otherwise	U.K. HLS - Understanding Society wave #10
Higher education	1 if completed level of education is 4–7 of U.K. education system, 0 otherwise	U.K. HLS - Understanding Society wave #10
Subjective view of financial situation	five-point Likert scale with the following dimensions: 1) living comfortably; 2) doing alright; 3) just getting by; 4) finding it quite difficult; and 5) finding it very difficult.	U.K. HLS - Understanding Society wave #10
NHS shielding category	1 if NHS told him/her that he/she is at severe risk of COVID-19 infection, 0 otherwise	COVID-19 Survey wave #1
Charitable donations	1 if respondent donates money to charity, 0 otherwise	U.K. HLS - Understanding Society wave #10
Non-proximity with non-cohabitating children	1 if respondent lives more 30 than minutes journey time of their children, 0 otherwise	U.K. HLS - Understanding Society wave #9
Gmobility index	Google mobility index obtained from the principal component analysis. It was normalized to lie between 0 (lowest bound) and 1 (highest bound)	Google mobility data
Pre-existing Poor Health Conditions (SAH)	1 if SAH is fair or poor, 0 otherwise	U.K. HLS - Understanding Society wave #10
Pre-existing Mental Health Conditions/ Psychological Distress 2019 (GHQ>=3)	1 if GHQ-12 Caseness items score measured in 2019 is greater or equal than 3 reflecting deteriorations in mental health, 0 otherwise.	U.K. HLS - Understanding Society wave #10
No friends living in local area	1 if the respondent has no friends living in local area, 0 otherwise.	U.K. HLS - Understanding Society wave #9
Who deals with formal care payments	1 if the respondent deals with formal care payments partly or entirely by herself, 0 otherwise.	U.K. HLS - Understanding Society wave #9

For our study, we considered the following categories: demographics, socioeconomic variables, and health conditions that existed before the COVID-19 outbreak. Among demographics, we included the respondent’s gender (1: male; 0: female), age, rural living (1: rural area; 0: urban area), area-level context captured with regional fixed effects (i.e., Wales, Scotland, Northern Ireland, and English region), and type of household categorized into single-household living *vs*. living with a partner. We also included an indicator of social capital and two COVID-related variables: one in the NHS Shielding category, and the other related to changes of individuals’ mobility due to COVID-19.

Among the socioeconomic variables, we included an indicator of respondents’ living standards that may influence the probability of psychological distress, the probability of accessing formal and informal care, and the respondents’ education level. Specifically, concerning the living standards, we included an indicator of respondents’ subjective views of their financial situation as measured by the question, “*How well would you say you yourself are managing financially these days?*” Responses were coded with a five-point Likert scale with the following dimensions: (1) living comfortably; (2) doing alright; (3) just getting by; (4) finding it quite difficult; and (5) finding it very difficult. Thus, the score ranged between 1 and 5 with a higher score indicating a worse financial situation. Concerning the education level, three levels were considered: (1) lower education (no qualifications or basic qualifications—i.e., level 1–2 in the U.K. education system); (2) medium education (level 3 in the U.K. education system or equivalent qualifications); and (3) higher education (i.e., levels 4–7 in the U.K. education system).

To account for respondents’ “needs” unrelated to the pandemic itself and the associated lockdown, we also included information on their health status before the outbreak (U.K. HLS wave #10). The health-related variables concerned an indicator of general health, the self-assessed health (SAH), and the presence of a pre-existing mental condition. The SAH is supported by literature that shows a strong predictive relationship between people’s self-rating of their health and mortality or morbidity (Idler and Benyamini, 1997, Kennedy et al., 1998). Moreover, the self-assessed health measurement correlates strongly with more complex health indices, such as functional ability or indicators derived from health service use (Undon and Elofsson, 2006). The following standard self-assessed health status question was asked: ‘*Would you say that in general your health is: 1) excellent, 2) very good, 3) good, 4) fair, 5) poor.*” Since the answers could not simply be scored (for example as 1, 2, 3, 4, 5) because the true scale will not be equidistant between categories (O’Donnell et al., 2008) according to previous literature (see, for instance, Balia and Jones, 2008; Di Novi, 2010; Di Novi, 2013), we dichotomized the multiple-category responses and constructed a binary indicator with a value of 1 if individuals reported that their health was fair or poor, and 0 otherwise (i.e., excellent, very good, or good). Pre-existing mental condition was identified using the GHQ-12 Caseness dummy indicator from U.K. HLS wave #10.

Concerning the indicator of social capital, we included a binary variable among the controls that takes value of 1 if respondents donated to a charity organization the year before the COVID-19 outbreak. Donating money to charity organizations is an indicator of social capital that we expect might influence informal care reception (and its disruption) and individuals’ psychological health (Dunn et al., 2008).

Among the regressors, we included a dummy variable that indicated whether respondents were in the NHS Shielding category. In March 2020, the U.K. government introduced a Shielded Patient List (SPL)- i.e., a record of clinically vulnerable patients thought to be at higher risk of severe COVID-19 complications and COVID-19-related death. Those patients on the SPL were sent a notification by the NHS or the Chief Medical Officer to encourage them to stay in their homes and keep away from the rest of the population for 12 weeks. In our study, the NHS Shielding category (Yes/No) is ascertained from the COVID-19 Survey on the basis of a self-reported answer to the following question: “*Have you received a letter, text or email from the NHS or Chief Medical Officer saying that you have been identified as someone at risk of severe illness if you catch coronavirus, because you have an underlying disease or health condition*?” We expected that belonging to the NHS Shielding category might have directly affected informal and formal care reception as well as older individuals’ mental health. Indeed, the elderly, especially those with cognitive decline and long-term conditions, need emotional support through informal networks and health professionals. As such, the lockdown might have created isolation and disruption of care along with a new set of challenges that could also affect other pre-existing health concerns, including mental health consequences (even though strict isolation was necessary to protect the elderly against the risks of the coronavirus). About 10% of our sample was notified as belonging to the NHS Shielding category as individuals extremely vulnerable to COVID-19.

Finally, we included an indicator of changes of individuals’ mobility due to COVID-19. We took advantage of a human mobility data set, the Google Covid-19 Mobility Report (GCMR) (Google LLC, 2021) that reports changes in the mobility of Google Maps users across different destination categories (e.g., supermarkets, pharmacies, workplaces, residential areas) with respect to the first two months of 2020 (pre-COVID-19 outbreak). This data set is public and available in a variety of countries.

We built a mobility index that combined different Google mobility categories into a single variable using two data sources: Understanding Society and the GCMR. Understanding Society considers 12 regions based on the Nomenclature of Territorial Units for Statistics (NUTS-1) Subdivision including Wales, Scotland, and Northern Ireland plus 9 regions in England (North East, North West, Yorkshire and The Humber, East Midlands, West Midlands, East, London, South East, and South West). We also used data on the total population in each region or the years 2015–2019 from the ONS (Office for National Statistics, 2020).

The GCMR provides daily mobility data for six location categories: residential, workplace, supermarket, and pharmacy (grocery), transit, retail, and parks (Google LLC, 2021). Data are reported as percentage variations in the number of visits or time spent in each category with respect to a pre-COVID-19 baseline period defined from January 3 to February 6, 2020. Google chooses this reference period, and thus it cannot be modified. To protect users’ privacy, absolute mobility values are not available.

Mobility data are available for each GCMR category for 108 sub-national regions (the GCMR’s variable is called sub_region_1), from February 15 (the first available date in the data set) to August 14, 2020. We aggregated the GCMR data by week (for consistency with Understanding Society’s questions on informal and formal care received and change in the care provision) and region (taking the weighted average across all counties in each region, with weights equal to their population sizes).

For each region analysed in our paper, we then extracted the most significant information from the different GCMR categories by merging them into a combined “Google mobility index” (see Basellini et al., 2021). In other words, we worked with two dimensions (categories and regions) simultaneously. We performed a principal component analysis (PCA) of the mobility data and extracted the first component for the region, which is identified as using the component with the largest proportion of explained variance as criteria. Accordingly, we built a Google mobility index (*Gmobility*). In constructing the index, we considered five location categories instead of six dimensions; specifically, we did not include the PCA residential category because it was missing too many values. The Google mobility index was standardized (see Basellini et al., 2021) for ease of interpretation.

The multivariate probit estimation was performed using the STATA 17 software and the use of the simulated maximum likelihood estimation method (see Cappellari and Jenkins, 2003).

## 4 Results and discussion

Table 2 shows a simple descriptive analysis that presents sample means and standard deviations for the variables used in the model (48% male; mean age: 72 years). Note the prevalence of psychological distress based on the GHQ‐12 Caseness scoring, which increased from 13.7% at the time of wave #10–26.4% at the peak in April of 2020. About 4% of respondents reported that they experienced informal care disruption, and approximately 3% reported formal care disruption (as previously stated). Approximately 21% of the respondents reported fair or poor health before the onset of the pandemic.

**Table 2:** Descriptive Statistics.

	Mean	SD
Mental Health Conditions/Psychological Distress (GHQ >= 3)	0.264	0.441
Formal Care Disruption	0.027	0.161
Informal Care Disruption	0.041	0.198
Age	72.19	5.446
Male	0.481	0.500
Rural	0.334	0.472
England	0.819	0.385
Wales	0.059	0.235
Scotland	0.089	0.285
Northern Ireland	0.033	0.177
Living with partner	0.746	0.435
Lower education	0.275	0.447
Medium education and other qualification	0.277	0.447
Higher education	0.448	0.497
Subjective view of financial situation	1.605	0.727
Charitable donations	0.825	0.380
Pre-existing Poor Health Conditions (SAH)	1.605	0.727
Non-proximity with non-cohabiting children	0.103	0.304
NHS shielding category	0.104	0.305
Pre-existing Mental Health Conditions/Psychological Distress 2019 (GHQ >= 3)	0.137	0.344
No friends living in local area	0.034	0.180
Who deals with formal care payments	0.079	0.270
Observations	3721	

Table 3 presents the results of the multivariate regression model with exclusion restrictions (the model without exclusion restrictions is included in the Appendix). Columns 1 and 2 report the estimated marginal effects for a disruption in informal care and formal care respectively, and Column 3 reports those respondents with psychological distress.

**Table 3:** Multivariate Probit Model—Estimated Marginal Effects.

	(1)	(2)	(3)
	Informal care disruption	Formal care Disruption	Mental Health Conditions/Psychological Distress (GHQ>=3)
Age	-0.002**	0.001***	-0.002
	(0.001)	(0.000)	(0.001)
Male	-0.021***	0.008	-0.104***
	(0.007)	(0.005)	(0.014)
Rural	0.000	-0.002	-0.004
	(0.007)	(0.006)	(0.015)
Wales	-0.025***	0.002	-0.057**
	(0.009)	(0.012)	(0.028)
Scotland	0.004	0.003	-0.008
	(0.011)	(0.010)	(0.024)
Northern Ireland	0.007	0.006	-0.029
	(0.020)	(0.016)	(0.039)
Living with partner	0.003	-0.003	-0.058***
	(0.007)	(0.006)	(0.017)
Medium and other education	0.021*	0.008	-0.012
	(0.011)	(0.007)	(0.018)
Higher education	0.019**	-0.003	0.045***
	(0.009)	(0.006)	(0.017)
Subjective view of financial situation	0.009**	0.006*	0.057***
	(0.004)	(0.003)	(0.010)
Pre-existing Poor Health Conditions (SAH)	0.015	0.033***	0.051***
	(0.010)	(0.008)	(0.019)
NHS shielding category	0.004	0.019**	0.014
	(0.011)	(0.009)	(0.023)
Charitable donations	-0.017**	-0.009	0.040**
	(0.006)	(0.007)	(0.018)
Gmobility Index	0.003	-0.000	0.014*
	(0.004)	(0.003)	(0.007)
Pre-existing Mental Health Conditions/ Psychological Distress 2019 (GHQ Caseness >= 3)	0.008	0.023**	0.264***
	(0.010)	(0.009)	(0.025)
Non- proximity with non-cohabiting children	0.013**		
	(0.006)		
No friends living in local area	0.038**		
	(0.016)		
Deals with care payments by herself		0.018**	
		(0.010)	
Informal care disruption			0.100***
			(0.037)
Formal care disruption			0.212***
			(0.051)
N	3721	3721	3721

Legend: * = 10% significance level, ** = 5% significance level, *** = 1% significance level

Starting with Column 1, the probability of informal care disruption decreases with age and for males. It is not significantly affected by the COVID-19 high-risk indicator (NHS Shielding category) for pre-existing mental health conditions, but instead increases with worsening pre-existing, self-reported general health conditions.

Table 3, Column 2 shows that formal care disruption is significantly and positively associated with variables that indicate a higher risk of adverse health outcomes if one contracts COVID-19. That is, the probability of formal care disruption increases with age and worsening pre-existing, self-reported health, and mental health conditions according to the COVID-19 high-risk indicator used in our study (i.e., being clinically extremely vulnerable to the COVID-19-NHS Shielding category). In general, these results confirm that older adults with pre-existing health conditions and for whom the consequences of catching the virus may be more serious faced the greatest social restrictions and stringent advice to stay at home. These adults were also more likely to experience a reduction of care, particularly in terms of community services. In such cases, formal care disruption was justified by the aim of protecting them from contracting COVID-19.^8^

As expected, during the pandemic, the likelihood of informal care disruption was higher when adult children did not live close to their parents and for respondents without friends in their local area (according to estimated marginal effects of 1.3% and 3.8%, respectively). Due to movement restrictions and lockdowns, older adults remained isolated in their homes with limited outside contact including those with non-cohabiting adult children and friends, which are considered critical factors in contributing to the spread of the virus (Arpino et al., 2021, Bayer C. , Kuhn M. (2020). Intergenerational ties and case fatality rates: A cross-country analysis. VoxEU.org. Retrieved from 〈https://voxeu.org/article/intergenerational-ties-and-case-fatality-rates〉). Table 3 also shows that the absence of any financial support received by respondents in paying for the costs of formal services increased the probability of formal care disruption by about 2%.^9^

Finally, the indicator of social capital, as expected, appears to have a negative influence on informal care disruption with a marginal effect of about 1.7%, given the association between social capital and the greater relationships within a community (Makridis and Wu, 2021).

In terms of socioeconomic status, perceived lower financial stability is associated with disruption in both informal and formal care even though the marginal effects are relatively low; moreover, according to our results, a higher education level positively influences informal care disruption only, with a marginal effect of about 2%. Arguably, a higher level of education raises awareness of the virus and may be positively associated with engagement in all types of preventive behaviors-including complying with stay-at-home rules. This implies a higher probability of in-person contact disruption and consequently the informal care provision particularly among the oldest population that is more vulnerable to COVID-19 infections (Li et al., 2020).

In reference to the structural equation (Column 3 in Table 3), our results show that formal and informal care disruption significantly increases the probability of psychological distress, with a marginal effect of about 10% and 21%, respectively. The disruption of routine community care provided by family members, friends, and especially those provided by paid caregivers or social services workers imposes a great psychological burden on older people. Informal and formal care are both important for older adults as they become more frail and dependent for care and support. However, formal care may better address the needs of those who struggle with multi-dimensional difficulties in their daily lives because of disabling physical and mental health conditions. Informal care behaves as a substitute for formal care in some circumstances only: for unskilled formal care. The substitutability tends to disappear as the level of disability of older persons increases (Bonsang, 2009). Arguably, disruption of formal care can compromise much more older adults psychological well-being through more unmet care needs (Allen et al., 2014).

Concerning the other variables included in the structural equation, our findings show that being male was associated with a lower probability of psychological distress during the COVID-19 outbreak with a marginal effect of around 10%. According to our results, while perceived lower financial stability increases the probability of suffering from psychological distress by about 6%, as expected, a higher education level seems to positively affect the probability of suffering from mental health conditions with a marginal effect of about 5%. A large part of the existing literature that has analysed the relationship between individuals’ mental health and education supports the protective role of education (see, among others, Feinstein, 2002; Chevalier and Feinstein, 2007; Crespo et al., 2014; Di Novi et al., 2021). Nevertheless, our results are in line with the most recent literature (Niedzwiedz et al., 2021, Daly et al., 2020, Pierce et al., 2020, Belo et al., 2020) that focused on mental health conditions following the COVID-19 outbreak. According to these contributions (that were mainly related to younger adults), groups most adversely affected in terms of psychological distress included women, younger adults, people from minorities groups, and those with a higher education level. The hypothesis is that the more educated groups were more likely to shift to remote work during the pandemic and, for some, this work was combined with home-schooling and resulted in an increased psychological burden (Niedzwiedz et al., 2021, Daly et al., 2020, Pierce et al., 2020). Concerning older individuals, further research is needed to shed light on this finding. Arguably, a higher level of education in this setting may proxy for an increasing awareness for older adults that they are at higher risk for severe morbidity and mortality from COVID-19, a circumstance that may also bring anxiety and readjustments in daily life and are likely stressful for this population (see Belo et al., 2020).

Respondents’ altruistic attitude, proxied by charitable donations in our study, contributes negatively to older adults’ psychological wellbeing This is consistent with recent research on altruism and mental health during the outbreak, suggesting that altruism does not serve as a protective mental health factor against the threat of COVID-19, as highly altruistic individuals are more likely to feel anxious and depressed due to their empathy towards infected people, and to the impossibility of helping others due to self-isolation regulations (Feng et al., 2020).

We estimate that a reduction of one standard deviation in the combined Google mobility index is associated with an increase of 1.4% in the probability of suffering from depression, which suggests that mobility limitations, as reflected by a decrease of movements, increases the likelihood of suffering from psychological distress.^10^

Finally, there exists a positive correlation between pre-existing health conditions, psychological distress (as measured by the SAH and GHQ-12 in 2019, respectively), and worsening mental health.

As previously discussed, we constructed a simultaneous equation model for three binary variables. The multivariate probit estimation allowed us to test for unobserved heterogeneity that may characterize the relationship between informal and formal care disruption and individuals’ psychological distress. The unobserved heterogeneity is captured by the correlation between the error terms from the single equation models. Table 4 shows the correlation coefficients for the full recursive model. The null hypothesis of exogeneity is rejected in only one case. According to our results, there exists a negative statistically significant correlation between the disturbance of the formal care disruption equation and the structural equation for individuals’ psychological distress-i.e., unobservable variables that increase the likelihood of depression and decrease the probability of disruption in formal care provisions. Arguably, the inability to access social support services due to COVID-19 contributes to worsening anxiety and depressive symptoms especially among the elderly affected by pre-existing mental health conditions. As such, the virus increases their demand of formal care support that in turn decreases the likelihood of formal care disruption.

**Table 4:** Correlation Coefficients from the Recursive Multivariate Probit Estimation (model with NHS Shielding category and treatment canceled).

	Informal Care Disruption	Formal Care Disruption	Mental Health Conditions/Psychological Distress (GHQ >= 3)
Informal Care Disruption	1	-0.094 (0.081)	-0.018 (0.054)
Formal Care Disruption		1	-0.096* (0.055)
Mental Health Conditions/Psychological Distress (GHQ >= 3)			1

Standard errors in parentheses

Legend: * = 10% significance level

## 5 Conclusions

In this paper, we investigated how informal and formal care disruption due to the COVID-19 outbreak have affected older people’s mental health. For the purposes of our analysis, we relied on individual-level data from the U.K. Household Longitudinal Study (U.K. HLS)-Understanding Society. We modeled the association between a disruption of formal and informal care received by the elderly and their mental health during the first wave of the COVID-19 pandemic by using a recursive simultaneous equation model for binary variables. According to our results, this disruption due to the COVID-19 emergency-and the aim of protecting the most vulnerable part of the population-has significantly affected older individuals’ psychological distress.

Although social distancing has reduced the rate at which infected individuals infect others, it has come at the cost of both an economic crisis as well as foregone benefits of physical social contacts that have profoundly reshaped LTC patterns. Social distancing has been necessary to protect older adults against the risk of severe infection and COVID-19-related death; however, such isolation may have created a new set of challenges affecting other pre-existing health concerns. It is well known that older people with unmet needs (as a potential consequence of informal and formal care disruption) cope with greater challenges and vulnerabilities correlated, in many instances, with poor mental health and anxiety (Komisar et al., 2005, Momtaz et al., 2012, He et al., 2015).

As lesson for future pandemics, the potential impact of the disruption of long-term care on older individuals’ mental health should be considered. Indeed, the possible benefits of mandatory lockdown in curbing the virus spread need to be carefully weighed against the potential psychological health costs. Successful use of isolation as a public health measure requires a realistic reduction in the negative effects associated with it, especially among more vulnerable groups.

One limitation of our data set is that it did not allow us to study possible differences of the disruption impacts related to territories, age groups, and gender. The sample size must be larger to implement heterogeneity tests. This is left for future research.

## Funding

None.

## CRediT authorship contribution statement

The interpretation and reporting of the results are the sole responsibility of the authors. All the authors contributed to the study conception, design, manuscript writing, and commented on/edited all drafts. The authors can be identified as guarantors for the overall content and interpretation of the results. The views expressed are purely those of the authors and may not in any circumstances be regarded as stating an official position of the European Commission.

## Declaration of Competing Interest

The authors report no declarations of interest.

## Figures and Tables

**Table A1:** Multivariate Probit Model – Estimated Marginal Effects.

	(1)	(2)	(3)
	Informal care disruption	Formal care Disruption	Mental Health Conditions/Psychological Distress (GHQ>=4)
Age	-0.002**	0.001***	-0.002
	(0.001)	(0.000)	(0.001)
Male	-0.021***	0.007	-0.104***
	(0.007)	(0.005)	(0.014)
Rural	0.000	-0.002	-0.004
	(0.007)	(0.006)	(0.015)
Wales	-0.025***	0.002	-0.057**
	(0.010)	(0.012)	(0.028)
Scotland	0.004	0.003	-0.008
	(0.011)	(0.010)	(0.024)
Northern Ireland	0.006	0.005	-0.029
	(0.020)	(0.016)	(0.039)
Living with partner	0.003	-0.003	-0.058***
	(0.007)	(0.006)	(0.017)
Medium and other education	0.021**	0.007	-0.012
	(0.011)	(0.007)	(0.018)
Higher education	0.019**	-0.003	0.045***
	(0.009)	(0.006)	(0.017)
Subjective view of financial situation	0.009**	0.006*	0.057***
	(0.004)	(0.003)	(0.010)
Pre-existing Poor Health Conditions (SAH)	0.015	0.033***	0.051***
	(0.010)	(0.008)	(0.019)
NHS shielding category	0.004	0.019**	0.014
	(0.011)	(0.009)	(0.023)
Charitable donations	-0.017*	-0.008	0.040**
	(0.010)	(0.007)	(0.018)
Proximity with non-cohabiting children	0.013**		
	(0.006)		
Gmobility Index	0.003	-0.000	0.014*
	(0.004)	(0.003)	(0.007)
Pre-existing Mental Health Conditions/ Psychological Distress 2019 (GHQ >= 4)	0.008	0.028***	0.264***
	(0.011)	(0.010)	(0.025)
No friends living in local area	0.037**		
	(0.016)		
Deals with care payments by herself		0.018*	
		(0.010)	
Informal care disruption			0.100***
			(0.037)
Formal care disruption			0.212***
			(0.051)
N	3721	3721	3721

**Table A2:** Multivariate Probit Model of the 12 items of the GHQ-12 – Estimated Marginal Effects.

	Concentration	Loss of sleep	Playing a useful role	Capable of making decisions	Constantly under strain
**Informal care disruption**	0.0339	0.0366	0.0609	-0.0179	0.0673*
	(1.13)	(1.26)	(1.67)	(−1.03)	(2.17)
**Formal care disruption**	0.270***	0.128**	0.113*	0.183***	0.106*
	(5.39)	(2.93)	(2.33)	(4.33)	(2.56)
**N**	3721	3721	3721	3721	3721
	**Problem overcoming difficulties**	**Enjoy day to day activities**	**Ability to face problems**	**Unhappy or depressed**	**Losing confidence**
**Informal care disruption**	0.0279	0.103*	0.0108	0.102**	-0.00716
	(1.22)	(2.51)	(0.55)	(2.91)	(−0.39)
**Formal care disruption**	0.136***	0.114*	0.108**	0.120**	0.130***
	(3.47)	(2.20)	(3.09)	(2.61)	(3.44)
**N**	3721	3721	3721	3721	3721
	**Believe worthless**	**General happiness**			
**Informal care disruption**	-0.0305**	-0.00327			
	(−3.24)	(−0.11)			
**Formal care disruption**	0.0249	0.120**			
	(1.08)	(2.65)			
**N**	3721	3721			

## Data Availability

Data will be made available on request.

## APPENDIX

### Sensitivity Analysis

In our main analysis, elderly’s psychological distress is measured by the 12-items Generalised Health Questionnaire (GHQ-12 Caseness), and respondents scoring 3 or more out of a possible total of 12 are considered at risk of anxiety and/or depression. First, we re-run the model setting the GHQ-12 Caseness threshold at 4, to identify higher intensities of mental health problems and how they are related to formal and informal care disruption (see Jones, 2021). Secondly, we re-run the model by considering as dependent variables binary indicators for each of the 12 items that comprise the GHQ-12 Caseness questionnaire.

### 1A. Different Threshold for the Generalised Health Questionnaire (GHQ-12)

As stated before, we rely on the same specification of the main model, with two reduced form (informal care and formal care disruptions) and one structural equation (mental health), while we move the threshold identifying mental health conditions at 4 symptoms. Results confirm those of the main analysis. According to our results (columns 1 and 2 of Table A1), elderly who live more than 30 min away from their children or who do not have any friend living in the same area are more likely to experience informal care disruption. Moreover, older adults who deal with care payments partly or totally by themselves, are more exposed to formal care disruption. Elderly with pre-existing health conditions are more affected by social restriction when it comes to formal care provision, thus being more likely to experience a reduction of care; whereas, this effects is no longer significant in the regression of informal care disruption. Finally, social capital decreases informal care disruption, while a perceived lower financial stability is associated with disruption in both forms of care.

With reference to the structural equation for psychological distress (column 3 in Table A1), results show that both informal and formal care disruption significantly raises the likelihood of experiencing psychological distress among elderly with higher intensities of mental health problems. Thus, our findings seem to support the hypothesis that, among the group of elderly people with more critical psychological conditions, the disruption of routine care provided by both informal caregivers as well as paid care workers or social services, due to lockdown policies, are concurrent causes of worsening of psychological distress.

See Appendix Tables A1 and A2.

### A. The Different Dimensions of the GHQ-12 Caseness

As second sensitivity analysis, we define a different outcome variable. We take binary indicators for each of the 12 questions that comprise the GHQ-12 Caseness questionnaire and re-run the model again (see Jones, 2021). Performing this evaluation allows us to further investigate the relation between each of the GHQ dimensions and formal and informal care disruption, identifying which are more related to one or the other dimension of care disruption.

The twelve dimensions of GHQ-12 are concentration, loss of sleep, playing a useful role, ability to make decisions, coping under strain, overcoming difficulties, enjoying activities, facing up problems, feeling depressed or unhappy, feeling worthless and general happiness. As explained in sub-Section 3.1, responses are answered on a four-category scale: “not at all”, “no more than usual”, “rather more than usual”, “much more than usual”. In order to create the binary indicator, for each dimension, we attribute the value 1 to the two categories indicating the most depressed states and 0 to the remaining two categories, reflecting better mental health (see Jones, 2021). We run again the multivariate probit model, substituting one at a time each binary indicator as outcome variable.

First of all, this analysis confirms that the model is well identified. First, in all regressions, distance from adult children and friends are statistically significant: the likelihood of informal care disruption is higher when adult children or friends do not live closer to the elderly, especially during the implementation of movement restrictions and lockdowns. Second, dealing with care payments is significant with positive sign in all regressions, suggesting that elderly are more likely to experience disruption of formal care when they have to afford the economic cost of the formal care services without any financial support.

Third, formal care disruption is statistically significant with positive sign in eleven out of twelve regressions, suggesting a positive, consistent relation between reduction or interruption of formal provision and worsening of the different dimensions of mental health. This evidence is not found only in the case of the item “Believe worthless”. On the other hand, informal care disruption is positively associated with the items “Constantly under strain” (at 5% level), “Enjoy day to day activities” (at 5% level), “Feeling unhappy or depressed” (at 5% level) and negatively related to “Believe worthless” (at 10% level). In other words, as we expected, elderly who suffer disruption of informal care and social distancing are more exposed to depression. According to our results, these four dimensions of psychological distress are the most affected by informal care disruption and are the items that drive the impact of informal care disruption on the aggregate GHQ-12 Caseness score.

## Footnotes

2 Several nursing homes, alongside hospitals, were, in many countries, epicenters of the COVID-19 crisis, with a large number of infections and even deaths, and were often unable to provide sufficient protection to the dependent elderly against health risks during the first waves of the pandemic (Achou et al., 2022; Di Novi and Santos, 2023).

3 A recursive model is a special case of a system of equations in which the endogenous variables are determined in sequence. Thus, the right-hand side of the reduced-form equations for the endogenous variables include exogenous variables only. The right-hand side of the structural equation includes the exogenous variables and the endogenous variables estimated by the reduced-form equations. The model’s development may be traced back to the pioneering work of Heckman (1978), and it is a common approach to deal with the endogeneity of binary dependent variables.

4 The GHQ-12 items refer to difficulties with sleep, concentration, problems in decision making, feeling overwhelmed, and other indicators of distress.

5 As a sensitivity check we also re-ran the model with a different threshold identifying mental health conditions using four symptoms. Results confirm those of the main analysis (see the Appendix).

6 In this question, “in need” meant those who had reported at least one health condition (i.e., asthma, arthritis, congestive heart failure, coronary heart disease, angina, heart attack or myocardial infarction, stroke, emphysema, chronic bronchitis, chronic obstructive pulmonary disease, cystic fibrosis, hypothyroidism or an under-active thyroid, cancer, diabetes, epilepsy, high blood pressure/hypertension, emotional, nervous or psychiatric problem, multiple sclerosis, H.I.V., chronic kidney disease, conditions affecting the brain and nerves, motor neurone disease, learning disability or cerebral palsy, problems with spleen, obesity, other long standing/chronic condition), or were having/waiting for treatment at the time of the interview (such as an operation or procedure planned, targeted therapy, tests/consultations).

7 In the first U.K. lockdown, which started on 23 March 2020, people were advised to stay home and to leave their home for essential reasons only, such as to attend essential work, acquire food or medicine, go to the hospital, exercise once a day, or provide care or assistance to a vulnerable person. Travel restrictions prevented people from traveling outside their local area, namely their village, their town, or a part of the city where they live with no specific indications of “travel distance”. So, in choosing the cut-off we followed the previous literature wherein a travel time of approximately 30 min can be considered a “short distance”—i.e., in principle, this allows frequent contact and supports exchanges between caregivers and receivers. Hence, geographical proximity was measured as a dummy variable: long distance (more than 30 min’ travel time) against short distance (less than 30 min’ travel time). We combined adult child caregivers who live less than 15 min away with those who live 15–30 min away (see Li et al., 2019; Thomas and Dommermuth, 2020; Artamonova and Syse, 2021). Among the control variables, we did not consider co-residing children, since the questions related to informal care refer to care and support received from family, neighbors, or friends who do not currently live in the same house/flat as the respondent.

8 Arguably, individuals with pre-existing poor health conditions were more likely to suffer from care disruptions because they had been using care before the pandemic.)

9 The inclusion restrictions required that the indicator variables of non-proximity with children and friends, and the variable that measures the absence of any support in affording the costs of social care, should be correlated with informal care and formal care disruptions respectively at the individual level. Our estimation results of the formal and informal care disruption equations (see Table 3) confirm that the estimated marginal effects are all statistically significant at the 5% level, indicating that the inclusion restrictions are clearly met (Li et al., 2021).

10 Apparently, according to our estimations, mobility limitations did not affect informal and formal care disruptions. In our view, this may be due to the fact that the mobility index we constructed was based on categories that are more indicative of general mobility as they are related to activity around workplaces, retail outlets and use of public transportation. Unfortunately, we cannot include the residential category because it was missing too many values as stated above. However, variations in residential areas mobility might be more indicative of decreased activity in locations around the home environment that could be more related to the informal and formal care reception.
